# Cognitive Enhancement of Repetitive Transcranial Magnetic Stimulation in Patients With Mild Cognitive Impairment and Early Alzheimer’s Disease: A Systematic Review and Meta-Analysis

**DOI:** 10.3389/fcell.2021.734046

**Published:** 2021-09-10

**Authors:** Ye Xie, Yunxia Li, Lu Nie, Wanting Zhang, Zijun Ke, Yixuan Ku

**Affiliations:** ^1^Center for Brain and Mental Well-Being, Department of Psychology, Sun Yat-sen University, Guangzhou, China; ^2^Department of Neurology, Tongji Hospital, School of Medicine, Tongji University, Shanghai, China; ^3^Peng Cheng Laboratory, Shenzhen, China

**Keywords:** Alzheimer’s disease, mild cognitive impairment, repetitive transcranial magnetic stimulation, cognitive function, meta-analysis

## Abstract

Repetitive transcranial magnetic stimulation (rTMS), a non-invasive brain stimulation technique, has been considered as a potentially effective treatment for the cognitive impairment in patients with mild cognitive impairment (MCI) and Alzheimer’s Disease (AD). However, the effectiveness of this therapy is still under debate due to the variety of rTMS parameters and individual differences including distinctive stages of AD in the previous studies. The current meta-analysis is aiming to assess the cognitive enhancement of rTMS treatment on patients of MCI and early AD. Three datasets (PubMed, Web of Science and CKNI) were searched with relative terms and finally twelve studies with 438 participants (231 in the rTMS group and 207 in the control group) in thirteen randomized, double-blind and controlled trials were included. Random effects analysis revealed that rTMS stimulation significantly introduced cognitive benefits in patients of MCI and early AD compared with the control group (mean effect size, 1.17; 95% CI, 0.76 - 1.57). Most settings of rTMS parameters (frequency, session number, stimulation site number) significantly enhanced global cognitive function, and the results revealed that protocols with 10 Hz repetition frequency and DLPFC as the stimulation site for 20 sessions can already be able to produce cognitive improvement. The cognitive enhancement of rTMS could last for one month after the end of treatment and patients with MCI were likely to benefit more from the rTMS stimulation. Our meta-analysis added important evidence to the cognitive enhancement of rTMS in patients with MCI and early AD and discussed potential underlying mechanisms about the effect induced by rTMS.

## Introduction

Alzheimer’s disease (AD) is the most common neurogenerative disorders, and is typically characterized by decline in cognition, behavior and activities of daily living. Mild cognitive impairment (MCI) is a prodromal stage of dementia, characterized by subjective cognitive deficits and objective memory impairment without impairment in daily activity (Petersen et al., 1999; Breton et al., 2019). The detrimental impact of Alzheimer’s disease and mild cognitive impairment on cognitive function in older adults has caused suffering of patients and burden on society. However, by now, clinical trials fail to develop drugs that would slow the dementia caused by Alzheimer’s disease. Therefore, the exploration of effective nonpharmacological intervention is critical to extend the current treatment of Alzheimer’s disease.

Recently, repetitive transcranial magnetic stimulation (rTMS) has received increasing attention for its prominence effect on the intervention for cognitive function in AD and MCI (Birba et al., 2017; Chang et al., 2018; Lin et al., 2019; Chou et al., 2020). Repetitive transcranial magnetic stimulation is a non-invasive method of brain stimulation in which a train of magnetic pulses is delivered to a specific target location of the brain. rTMS could facilitate neural coactivation and change the synaptic strength, and thus, rTMS is able to modulate the activity in cortical areas or connectivity in related networks and influence the synaptic neuronal activities including long-term potentiation, which is related to the learning and memory processes (Tegenthoff et al., 2005; Luber and Lisanby, 2014). rTMS involves trains of TMS pulse with various frequencies and intensities. It has been reported that high frequencies (higher than 5 Hz) would increase cortical excitability and low frequencies (lower than 1 Hz) would suppress cortical excitability (Maeda et al., 2000).

A series of literature has suggested the positive effects of rTMS on AD patients and with the growing body of the rTMS studies in MCI and AD recently, several meta-analyses have investigated the effects of rTMS in older adults with MCI or AD and demonstrated a beneficial effect of rTMS on the cognitive function of patients (Lin et al., 2019; Chou et al., 2020; Wang et al., 2020; Jiang et al., 2021). However, most of them included patients within different stages of AD and resulted in large variety of pretreatment cognitive capability (Lin et al., 2019; Wang et al., 2020). Few studies have focused on the patients with MCI (Jiang et al., 2021). Previous studies have declared that the synaptic plasticity and cortical excitability, which play important roles in the rTMS-underlying mechanism, might be impaired in the early course of AD, even in MCI (Nardone et al., 2014; Koch et al., 2020). Given this, putting subjects with different stage of AD together may result in imprecise evaluation of the cognitive benefit of rTMS in specific stage of AD, especially in the early AD and MCI. *Ruthurford et al.* has reported the more marked cognitive benefits in early AD after rTMS stimulation and emphasized the importance of applying rTMS on early stage of AD (Rutherford et al., 2015). Thus, studying the effect of rTMS on the critical transitional stage, the MCI and early stage of AD, would extend our understanding in how to prevent the progression to dementia. Meanwhile, the condition of patients before receiving the rTMS has been reported to contribute to the variability of the rTMS-induced cognitive improvement (Anderkova et al., 2015) but such effect was barely investigated by previous meta-analyses. Besides, Some of the meta-analyses included studies without a randomized, controlled design, which cannot provide strong confidence about effect the of rTMS on the cognitive function in patients (Lin et al., 2019). Furthermore, some analyses included studies with less than 5 rTMS sessions, which could not provide enough stimulation for a complete rTMS protocol which can be applied for therapy (Chou et al., 2020). Previous rTMS studies mainly utilized high-frequency stimulation in AD patients to induce cortical excitability, and the most common frequency is 10 Hz and 20 Hz. DLPFC has been used as the typical stimulation site, and some studies applied rTMS over DLPFC only while some studies combined DLPFC with multiple sites over parietal and temporal cortex. The treatment duration was also distinctive in different studies, and most clinical trials utilized 20 to 48 sessions with 5 sessions per week. The therapeutic schedule and parameter design, such as target site and treatment course, and also the post-treatment effect of the intervention still require further investigation to develop more efficient intervention protocol. Therefore, the aim of this systematic review and meta-analysis was to provide up-to-date evidence on the effects of rTMS treatment on cognitive function in patients with MCI and early stage of AD based on a series of randomized, double-blind and controlled studies.

## Method

### Search Strategies

Databases of peer-reviewed literature were systematically searched on PubMed, Web of Science and CNKI for manuscripts about studies of the effect of rTMS on MCI or early AD, published online before March, 29, 2021. The English keywords used for the database searches were “mild cognitive impairment”, “MCI”, “Alzheimer’s Disease”, “AD”, “transcranial magnetic stimulation”, “repetitive transcranial magnetic stimulation”, “TMS”, “rTMS”. The Chinese keywords were “Qingdurenzhisunhai” (轻度认知损害), “Qingdurenzhizhangai” (轻度认知障碍), “Aerzihaimozheng” (阿尔兹海默症), “Aerzihaimobing” (阿尔兹海默病), “Qingdurenzhigongnengzhangai” (轻度认知功能障碍), “Chongfujingluciciji” (重复经颅磁刺激), “Jingluciciji” (经颅磁刺激). The reference lists of identified articles were checked for other potential studies.

### Inclusion and Exclusion Criteria

The inclusion criteria for the primary relevant published studies were: (1) human search; (2) randomized controlled studies investigating the effects of rTMS treatment of the cognitive function of patients with MCI or early AD (mean score of ADAS-Cog < 25 or of MMSE/MoCA > 19); cognitive impairment was caused by AD; (3) rTMS was used as the sole treatment measure or in combination with other treatments, and compared with sham-rTMS, pharmacological treatments or cognitive training; (4) continuously stimulate for at least 20 sessions; (5) sufficient original data was provided. The exclusion criteria were: (1) cognitive impairment caused by other disease; (2) duplicate publications; (3) articles published in non-English and non-Chinese languages; (4) articles published in the form of case report, comment, letter, review, abstract or patent.

### Data Extraction

Two researchers (YX, LN, and WZ) participated in extracting data from each included study, comparing their results and discussing to reach a consensus if there were disagreements. Extracted data included basic study information (author, year, and study design), sample size, sample characteristics (age, gender, education, disease type, disease duration), rTMS protocol (number of sessions, frequency, stimulation site), statistical data of the score of cognitive performance, the timing of outcome measurements, dropout rate and adverse effects. Authors of the original article were contacted if the information was unclear or insufficient.

### Evaluation of Risk of Bias

The risk of bias was assessed using the method recommended by the Cochrane Collaboration in Rev-man. The following characteristics were evaluated: (a) adequacy of sequence generation; (b) allocation concealment; (c) use of blinding; (d) how incomplete outcome data (dropouts) were addressed; (e) evidence of selective outcome data reporting; and (f) other potential risks that may harm the validity of the study. The risk of bias for each domain was graded as low, high, or unclear.

### Data Analysis

The meta-analysis was conducted with meta package in R (Balduzzi et al., 2019). We used standardized mean difference (SMD, also known as Hedges’ g given the small sample of the included studies) to express the effect size of rTMS on cognitive functions. The effect size and 95% confident interval (CI) were calculated according to the differences between poststimulation evaluations or changes relative to the baseline. With the studies which provided the outcome of pre- and post-stimulation and also the *p*-value or *t*-value of the paired sample *t*-test for each group, we calculated the change relative to the baseline for the group with the formulas below:


$$\begin{array}{c} M_{\mathrm{change}}=M_{\mathrm{prestimulation}}-M_{\mathrm{poststimulation}}\\ \;SD_{\mathrm{change}}=M_{\mathrm{change}}/(t/\sqrt{n}) \end{array}$$

*M*_*change*_ is the change score and *SD*_*change*_ is the standard deviation of change score. *t* is the *t*-value of paired sample *t*-test, and *n* is the sample size of the group.

The heterogeneity across effect sizes was assessed with *Q*-statistics and the *I*^2^ index, which is useful for assessing consistency between studies. When heterogeneity was found by *Q*-statistics or when I2 > 50%, a random effects model was applied. If not, a fixed effects model was used. If the effect size were reported from different subgroups of patients with different severity of AD within a single study, the data were included as independent units in the meta-analysis.

To address the possibility of publication bias, Egger’s test was conducted and a *p*-value < 0.05 indicated a publication bias. Due to the heterogeneity of cognitive measures included in each study, sensitivity analysis was also conducted to test whether our results would have differed if we omitted the included studies one by one. Subgroup analyses were performed separately according to cognitive domains, stimulus site, stimulus frequency, treatment course, disease duration and time points.

## Results

### Search and Selection of Studies

**FIGURE 1:**
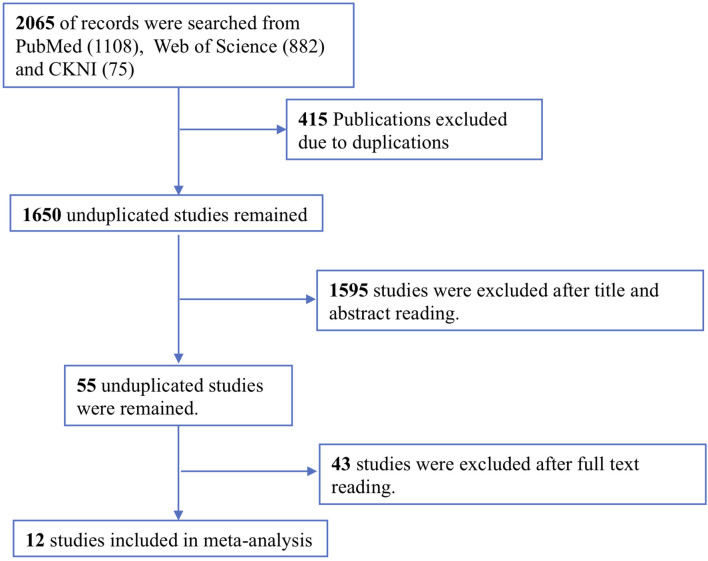
Flowchart of the literature search and screening processes.

The study selection process is shown in Figure 1. A total of 2065 potentially relevant studies were identified from two English and one Chinese database using relevant search strategies. Of this relevant studies, 415 duplicates were removed. During the title and abstract screening phase of the remaining 1650 studies, an additional 1595 studies were removed. Finally, after reading the full texts of the remaining 55 articles, 43 articles were excluded, thus twelve studies were included in this meta-analysis.

### Characteristics of the Induced Studies

Table 1 shows the characteristics of the twelve studies included in this meta-analysis, comprising a total of 438 participants (231 in the rTMS group and 207 in the control group) (Han et al., 2013; Rabey et al., 2013; Yang et al., 2014; Sun and Ma, 2015; Lee et al., 2016; Zhao et al., 2017; Wen et al., 2018; Zhang et al., 2019; Bagattini et al., 2020; Wen et al., 2020; Zhu et al., 2020; Yuan et al., 2021). Six studies were in English (Rabey et al., 2013; Lee et al., 2016; Zhao et al., 2017; Zhang et al., 2019; Bagattini et al., 2020; Yuan et al., 2021), and the remaining trials were in Chinese. Participants in seven studies were diagnosed as MCI (Han et al., 2013; Yang et al., 2014; Sun and Ma, 2015; Wen et al., 2018, 2020; Zhu et al., 2020; Yuan et al., 2021), while the participants in the rest five studies were diagnosed as mild to moderate AD. Among these five studies, two studies reported the behavioral outcomes for mild AD and moderate AD, respectively (Lee et al., 2016; Zhao et al., 2017). However, in the moderate subgroup of *Lee* et al., the score of ADAS-Cog was > 25 and the score of MMSE < 19, thus, only the mild subgroup of *Lee* et al. was included in the meta-analysis. Table 2 shows the characteristics of rTMS intervention in the included studies. Seven studies applied rTMS stimulation on unilateral/bilateral dorsolateral prefrontal cortex (DLPFC) only (Han et al., 2013; Yang et al., 2014; Wen et al., 2018, 2020; Bagattini et al., 2020; Zhu et al., 2020; Yuan et al., 2021), and the rest of the studies applied rTMS stimulation on multiple sites, including (a) parietal lobule and temporal lobule (Zhao et al., 2017), (b) left DLPFC and prefrontal cortex (PFC) (Sun and Ma, 2015), (c) left DLPFC and left lateral temporal lobe (LTL) (Zhang et al., 2019) and (d) Broca’s area, Wernicke’s area, bilateral DLPFC and bilateral parietal somatosensory association (pSAC) (Rabey et al., 2013; Lee et al., 2016). Seven studies utilized a frequency of 10 Hz (Rabey et al., 2013; Lee et al., 2016; Wen et al., 2018, 2020; Zhang et al., 2019; Zhu et al., 2020; Yuan et al., 2021), one studies used a frequency of 15 Hz (Sun and Ma, 2015) and four studies used a frequency of 20 Hz (Han et al., 2013; Yang et al., 2014; Zhao et al., 2017; Bagattini et al., 2020). The number of intervention sessions ranged from 20 to 48, and the timing for post-treatment assessment ranged from one to two month after treatment end. One study combined rTMS with electroacupuncture (Wen et al., 2020) and five studies combined rTMS with cognitive training (Rabey et al., 2013; Sun and Ma, 2015; Lee et al., 2016; Zhang et al., 2019; Bagattini et al., 2020).

**TABLE 1:** Demographic characteristics of the included trials.

References	Interventions	Final Sample Size (M/F)	Age (Year)	Education (Year)	Disease	Disease duration (Year)
Han et al., 2013	T: active rTMS	20 [original 22 (8/14)]	66.5 ± 5.02	11.35 ± 2.23	MCI	\
	C: sham rTMS	18 (6/12)	66.7 ± 5.25	11.11 ± 3.16		
Rabey et al., 2013	T: active rTMS	7 (5/2)	72.6 ± 8.9	\	mild to moderate AD	\
	C: sham rTMS	8 (5/3)	75.4 ± 9.07			
Yang et al., 2014	T: active rTMS	18 (8/10)	65.4 ± 5.6	\	MCI	1.57 ± 0.78
	C: sham rTMS	15 (7/8)	63.4 ± 8.2			1.83 ± 0.47
Sun and Ma, 2015	T: active rTMS + cognitive training	39, [original 40 (23/17)]	65.4 ± 5.6	\	MCI	1.57 ± 0.78
	C: sham rTMS	40 (20/20)	63.4 ± 8.2			1.83 ± 0.47
Lee et al., 2016	T: active rTMS	18 (8/10)	72.1 ± 7.6	9.9 ± 4.8	mild to moderate AD	\
	C: sham rTMS	8 (3/5)	70.3 ± 4.8	9.9 ± 3.7		
Zhao et al., 2017	T: active rTMS	17 (7/10)	69.3 ± 5.8	4.8 ± 1.9	mild to moderate AD	\
	C: sham rTMS	13 (6/7)	71.4 ± 5.2	4.9 ± 3.5		
Wen et al., 2018	T: active rTMS	23 (14/9)	64.17 ± 5.21	\	aMCI	4.06 ± 2.12
	C: sham rTMS	22 (10/12)	65.91 ± 4.93			4.38 ± 2.14
Zhang et al., 2019	T: active rTMS + cognitive training	15 (3/12)	69.00 ± 8.19	12.40 ± 2.06	mild to moderate AD	3.53 ± 1.81
	C: sham rTMS	13 (3/10)	68.54 ± 7.93	11.85 ± 2.38		3.62 ± 2.02
Wen et al., 2020	T: active rTMS+electro-acupuncture	22 (11/11)	64.59 ± 5.78	\	aMCI	4.23 ± 2.04
	C: electro-acupuncture	23 (9/14)	65.96 ± 4.82			4.24 ± 1.83
Bagattini et al., 2020	T: active rTMS + cognitive training	27 (17/10)	73.56 ± 4.91	8.85 ± 3.91	MCI or mild to moderate AD	1.94 ± 0.74
	C: sham rTMS	23 (12/11)	73.35 ± 1.09	7.91 ± 0.67		1.67 ± 1.26
Zhu et al., 2020	T: active rTMS	13 (7/6)	64.17 ± 5.21	\	aMCI	3.58 ± 2.31
	C: sham rTMS	12 (5/7)	65.91 ± 4.93			4.31 ± 2.17
Yuan et al., 2021	T: active rTMS	12 (6/6)	65.08 ± 4.89	11.83 ± 2.37	aMCI	4.25 ± 2.26
	C: sham rTMS	12 (6/6)	64.67 ± 4.77	11.33 ± 2.15		3.50 ± 2.23

*M, male; F, female. MCI, mild cognitive impairment; AD, Alzheimer’s disease.*

**TABLE 2:** Description of rTMS intervention in the included studies.

References	Interventions	Sessions	RMT (%)	Frequency (Hz)	Stimulating Site	stimulus pulses	with COG	Follow-up Assessment	Drop Out	Adverse Effect
Han et al., 2013	T: active rTMS	5 sessions/week for 8 weeks	80	20	bilateral DLPFC	600 for each region	No	\	2	4
	C: sham rTMS								0	3
Rabey et al., 2013	T: active rTMS	5 sessions/week for 6 weeks, 2 sessions/week for 3 months	90-110	10	Broca; Wernicke; R/L DLPFC; R/L pSAC	1300	Yes	\	1	0
	C: sham rTMS								2	0
Yang et al., 2014	T: active rTMS	5 sessions/week for 8 weeks	80	20	bilateral DLPFC	\	No	\	0	2
	C: sham rTMS								3	1
Sun and Ma, 2015	T: active rTMS + cognitive training	6 sessions/week for 8 weeks	80-110	15	L DLPFC/L PFC	\	Yes	\	1	2
	C: sham rTMS								0	0
Lee et al., 2016	T: active rTMS	5 sessions/week for 6 weeks	90-110	10	Broca; Wernicke; R/L DLPFC; R/L pSAC	1200	Yes	6 weeks after treatment end	0	0
	C: sham rTMS								1	0
Zhao et al., 2017	T: active rTMS	5 sessions/week for 6 weeks	\	20	P3/P4, T5/T6	\	No	6 weeks after treatment end	0	2
	C: sham rTMS								0	1
Wen et al., 2018	T: active rTMS	5 sessions/week for 4 weeks	80	10	L DLPFC	400	No	4 weeks after treatment end	1	0
	C: sham rTMS								1	0
Zhang et al., 2019	T: active rTMS + cognitive training	5 sessions/week for 4 weeks	100	10	L DLPFC and L LTL	1000	Yes	4 weeks after treatment end	0	0
	C: sham rTMS								2	0
Wen et al., 2020	T: active rTMS+electro-acupuncture	5 sessions/week for 4 weeks	80	10	L DLPFC	400	No	4 weeks after treatment end	0	2
	C: electro-acupuncture								1	0
Bagattini et al., 2020	T: active rTMS + cognitive training	5 sessions/week for 4 weeks	100	20	L DLPFC	2000	Yes	8 weeks after treatment end	0	0
	C: sham rTMS								0	0
Zhu et al., 2020	T: active rTMS	5 sessions/week for 4 weeks	80	10	L DLPFC	400	No	\	0	0
	C: sham rTMS								0	0
Yuan et al., 2021	T: active rTMS	5 sessions/week for 4 weeks	80	10	L DLPFC	400	No	4 weeks after treatment end	1	3
	C: sham rTMS								1	0

*RMT, resting motor threshold; COG, cognitive training; L, left; R, right; DLPFC, dorsolateral prefrontal cortex; PFC, prefrontal cortex; LTL, lateral temporal lobe; pSAC, parietal somatosensory association.*

For the outcome measures, different cognitive assessments were used to assessed same cognitive domain within a study or among studies. Measures for global cognitive function included Mini-Mental State Examination (MMSE) (Yang et al., 2014; Bagattini et al., 2020), Montreal Cognitive Assessment (MoCA) (Han et al., 2013; Sun and Ma, 2015; Wen et al., 2018, 2020; Zhu et al., 2020; Yuan et al., 2021) and AD Assessment Scale-cognitive subscale (ADAS-Cog) (Rabey et al., 2013; Lee et al., 2016; Zhao et al., 2017; Zhang et al., 2019). For the memory domain, the assessment included Rey Auditory Verbal Learning Test (RAVLT) (Bagattini et al., 2020), episodic memory (Han et al., 2013), World Health Organization University of California-Los Angeles (Zhao et al., 2017), Auditory Verbal Learning Test (WHO-UCLA AVLT) (Bagattini et al., 2020), the delay memory subscale of MoCA (Sun and Ma, 2015) and the memory sub-domain of Addenbrooke’s Cognitive Examination III (ACE-III) (Zhang et al., 2019). Trial Making Test-A (TMT-A) (Bagattini et al., 2020), alternating trial making (Han et al., 2013), attention subscale of MoCA (Sun and Ma, 2015) and attention sub-domain of ACE-III (Zhang et al., 2019) were used to assess the executive function and attention domain. Semantic verbal fluency (Bagattini et al., 2020), language subscale of MoCA (Sun and Ma, 2015), language sub-domain of ACE-III (Zhang et al., 2019) were used for the measurement of language ability.

Summary of effect sizes for global cognitive function and different cognitive domains assessed immediately after the treatment end were presented by Tables 3, 4. Effect sizes of three studies were calculated according to the provided change relative to the baseline (Rabey et al., 2013; Zhang et al., 2019; Yuan et al., 2021). Effect sizes of seven studies were calculated according to the poststimulation evaluations (Han et al., 2013; Yang et al., 2014; Sun and Ma, 2015; Wen et al., 2018, 2020; Zhu et al., 2020; Bagattini et al., 2020). Two studies provided the pre- and post-stimulation outcomes and statistical results of paired sample *t*-test for each group, thus the change relative to the baseline was calculated and two set of effect size were calculated with change relative to the baseline and poststimulation evaluation, respectively (Lee et al., 2016; Zhao et al., 2017). The effect sizes for global cognitive function assessed sometime after the treatment end were also calculated for the analysis to test the post-treatment effect of rTMS on cognitive function and the summary of the effect sizes was presented by Table 5.

**TABLE 3:** Summary of the effect sizes for global cognitive function.

No	Author	Outcome	Data Type	Nstim/Ncon	Mstim/Mcon	SDstim/SDcon	Hedges’ g	Lower	Upper
1	Han et al., 2013	MoCA	Poststimulation	20/18	28.28/23.17	2.02/2.79	2.0721	1.2771	2.867
2	Rabey et al., 2013	ADAS-Cog	Change	7/8	3.76/0.47	3.49/3.34	0.908	–0.1635	1.9796
	Yang et al., 2014	MMSE	Poststimulation	18/15	28.6/26.7	1.4/1.7	1.2015	0.4547	1.9484
3	Sun and Ma, 2015	MoCA	post	39/40	27.42/25.39	2.03/1.65	1.0881	0.615	1.5613
4	Lee et al., 2016 (mild)	ADAS-Cog	Change	13/6	5.46/2.66	8.28/7.02	0.3373	–0.6366	1.3111
			Poststimulation	13/6	16.31/18.17	6.4/4.54	0.3004	–0.6721	1.2729
5	Zhao et al., 2017 (mild)	ADAS-Cog	Change	12/8	4.2/4	6.40/7.07	0.4021	–0.5019	1.3061
			Poststimulation	12/8	16.4/20.3	4.4/5.6	0.762	–0.1659	1.69
6	Zhao et al., 2017 (moderate)	ADAS-Cog	Change	5/5	3.5/3.3	8.78/9.94	0.0193	–1.2204	1.2589
			poststimulation	5/5	20.3/24.2	6.5/8.6	0.4621	–0.7976	1.7218
7	Wen et al., 2018	MoCA	Poststimulation	23/22	25.09/21.73	1.08/1.35	2.7072	1.8913	3.5231
8	Zhang et al., 2019	ADAS-Cog	Change	15/13	3.37/0.84	2.59/2.49	1.001	0.2108	1.7913
9	Wen et al., 2020	MoCA	Poststimulation	22/23	25.55/23.74	1.34/1.84	1.1009	0.4722	1.7296
10	Bagattini et al., 2020	MMSE	Poststimulation	27/23	24.33/22.88	2.38/3.65	0.4712	–0.0928	1.0352
	Zhu et al., 2020	MoCA	Poststimulation	13/12	25.09/21.73	1.08/1.35	2.6707	1.5745	3.7669
	Yuan et al., 2021	MoCA	Change	12/12	2.25/0.25	1.86/1.48	1.1489	0.2808	2.0170

*Nstim/Mstim/SDstim, number of subjects/mean/standard deviation of stimulation group; Ncon/Mcon/SDcon, number of subjects/mean/standard deviation of control group; MoCA, Montreal Cognitive Assessment; MMSE, Mini-Mental State Examination; ADAS-Cog, AD Assessment Scale-cognitive subscale.*

**TABLE 4:** Summary of the effect sizes for different cognitive domains.

No	Author	Outcome	Data Type	Nstim/Nsham	Mstim/Msham	SDstim/SDsham	Hedges’ g	Lower	Upper
**Memory**									
1	Han et al., 2013	Epsodic memory	Poststimulation	20/18	7.73/6.28	2.45/1.63	0.675	0.0196	1.3304
2	Sun and Ma, 2015	Delay-memory subscale of MoCA	Poststimulation	39/40	3.71/3.09	0.72/0.14	1.1909	0.7116	1.6702
3	Zhao et al., 2017 (mild)	WHO-UCLA AVLT	Change	12/8	2.3/0.8	8.40/9.31	0.1639	–0.7323	1.0601
			Poststimulation	12/8	37.9/36.6	6.5/6.7	0.1893	–0.7074	1.086
4	Zhao et al., 2017 (moderate)	WHO-UCLA AVLT	Change	5/5	3/3.3	7.52/8.14	–0.0346	–1.2743	1.2051
			Poststimulation	5/5	33.5/33.9	2.3/5.4	–0.0871	–1.3274	1.1533
5	Zhang et al., 2019	Memory subscale of ACE-III	Change	15/13	3.87/0.29	0.82/1.07	1.029	0.2361	1.8219
6	Bagattini et al., 2020	RAVLT	Poststimulation	27/23	6.67/5.51	3.0/2.94	0.3841	–0.1773	0.9455
**Attention**									
7	Han et al., 2013	Alternating Trial Making	Poststimulation	20/18	61.10/74.94	25.62/17.63	0.6101	–0.0419	1.2621
8	Sun and Ma, 2015	Attention subscale of MoCA	Poststimulation	39/40	5.71/5.04	0.61/0.93	0.8414	0.3809	1.302
9	Zhang et al., 2019	Attention subscale of ACE-III	Change	15/13	2.19/0.07	0.44/0.49	1.2324	0.4187	2.0461
10	Bagattini et al., 2020	TMT-A	Poststimulation	27/23	52.85/49.05	54.45/26.99	–0.0849	–0.6413	0.4715
**Language**									
11	Sun and Ma, 2015	Language subscale of MoCA	Poststimulation	39/40	3.12/2.91	0.19/0.12	1.3123	0.8252	1.7994
12	Zhang et al., 2019	Language subscale of ACE-III	Change	15/13	2.31/1	0.89/0.92	0.3896	–0.3605	1.1397
13	Bagattini et al., 2020	Semantic verbal fluency	Poststimulation	27/23	32.11/29.17	9.53/7.11	0.3402	–0.2201	0.9004

*MoCA, Montreal Cognitive Assessment; WHO-UCLA AVLT, World Health Organization University of California-Los Angeles Auditory Verbal Learning Test; ACE-III, Addenbrooke’s Cognitive Examination III; RAVLT, Rey Auditory Verbal Learning Test; TMT-A, Trial Making Test-A.*

**TABLE 5:** Summary of the effect sizes for global cognitive function of the post-treatment assessments.

No	Author	Outcome	Data Type	Nstim/Nsham	Mstim/Msham	SDstim/SDsham	Hedges’ g	Lower	Upper	PTE (month)
1	Lee et al., 2016 (mild)	ADAS-Cog	Change	13/6	6.85/3.5	7.2/3.46	0.5052	–0.4766	1.4871	1.5
			Poststimulation	13/6	14.92/17.33	7.43/4.93	0.339	0.6349	1.3129	
2	Zhao et al., 2017 (mild)	ADAS-Cog	Change	12/8	6.4/2.3	7.89/7.97	0.4957	–0.4131	1.4046	1.5
			Poststimulation	12/8	14.2/19.4	6.8/6.8	0.7324	–0.1931	1.6578	
3	Zhao et al., 2017 (moderate)	ADAS-Cog	Change	5/5	4.9/4	8.73/9.8	0.0876	–1.1527	1.3279	1.5
			Poststimulation	5/5	18.9/23.5	2.3/5.4	0.5429	–0.7244	1.8102	
4	Wen et al., 2018	MoCA	Poststimulation	23/22	24.26/21.73	1.28/1.51	1.7792	1.0852	2.4732	1
5	Zhang et al., 2019	ADAS-Cog	Change	15/13	3.52/1.54	1.90/2.27	0.9602	0.1736	1.7467	1
6	Wen et al., 2020	MoCA	Poststimulation	22/23	24.91/22.35	1.11/1.47	1.9249	1.2139	2.6358	1
7	Bagattini et al., 2020	MMSE	Poststimulation	27/23	24.16/22.8	2.36/3.91	0.4228	–0.1397	0.9853	2
8	Yuan et al., 2021	MoCA	Change	12/12	1.25/-0.42	1.48/1.83	0.9689	0.1198	1.8179	1

*Nstim/Mstim/SDstim, number of subjects/mean/standard deviation of stimulation group; Ncon/Mcon/SDcon, number of subjects/mean/standard deviation of control group; MoCA, Montreal Cognitive Assessment; MMSE, Mini-Mental State Examination; ADAS-Cog, AD Assessment Scale-cognitive subscale.*

### Research Quality

**FIGURE 2:**
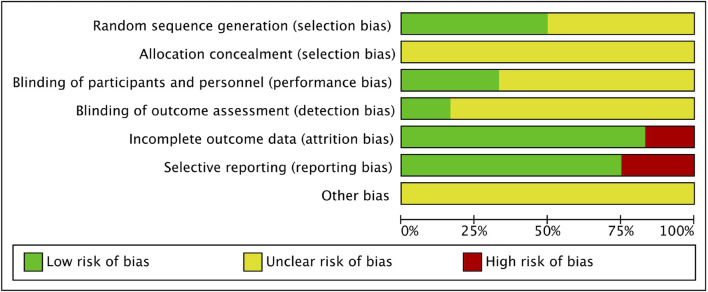
Risk of bias graph.

The summary of the risk of bias of the included studies is shown in Figure 2. All studies declared random allocation, but only six Studies described the method used to generate the random sequence in detail and were rated as “low risk.” All studies declared double-blind, but only four studies mentioned the participants and researchers were double-blinded, thus performance bias was rated as “low risk.” The risk of attrition bias was rated as “high risk” because the research data were incomplete (due to drop-out) without enough details for two studies. The reporting bias of three studies were rated as “high risk” due to reporting their results selectively. Studies with unclear information were rated as “unclear risk”.

### Meta-Analysis of Treatment Effect

#### Global Cognitive Function

**FIGURE 3:**
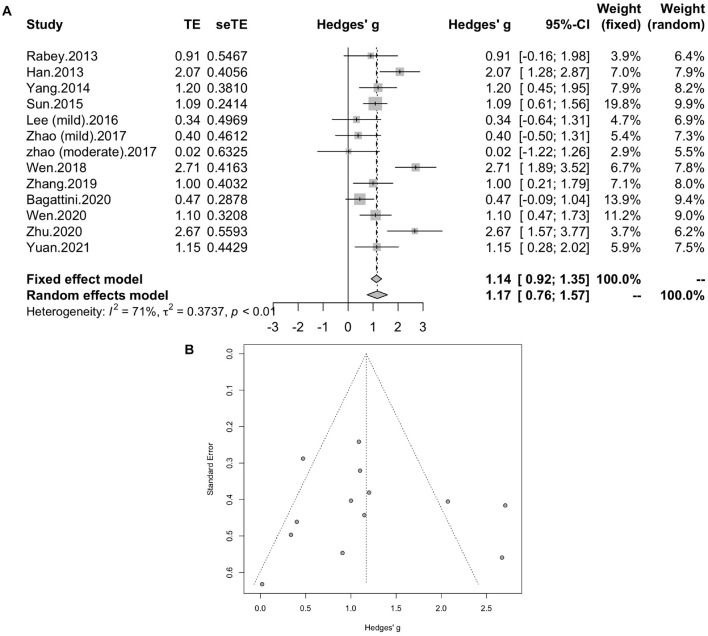
**(A)** Forest plot: mean differences in effect of rTMS on different cognitive domain in patients with MCI or early AD with 95% CI. **(B)** Funnel plot for the publication bias of global cognitive function.

All of the twelve studies, thirteen trials assessed the effects of rTMS on global cognitive ability. The heterogeneity of the included studies was high (I2 = 70.8%, *p* < 0.0001), so a random-effect model was used for the meta-analysis. The results demonstrated that rTMS treatment significantly improved the global cognitive function in the active rTMS group with a statistically significant mean effect size of 1.17 (95% CI, 0.76 - 1.57, *p* < 0.0001, Figure 3A) when compared to the control group. Egger’s test was used to test the publication bias and revealed an unsignificant asymmetry (*p* = 0.77, funnel plot in Figure 3B). Because of the high heterogeneity, sensitivity analysis was conducted and omitting the studies one by one didn’t alter the significance of effect size. Such results still remained significant by replacing the effect size of study *Zhao* et al. and study *Lee* et al. which is calculated with poststimulation evaluations (mean effect size 1.22, 95% CI: 0.83 – 1.61, *p* < 0.0001, Supplementary Figure 1).

#### Subgroup Analyses of Global Cognitive Function

**FIGURE 4:**
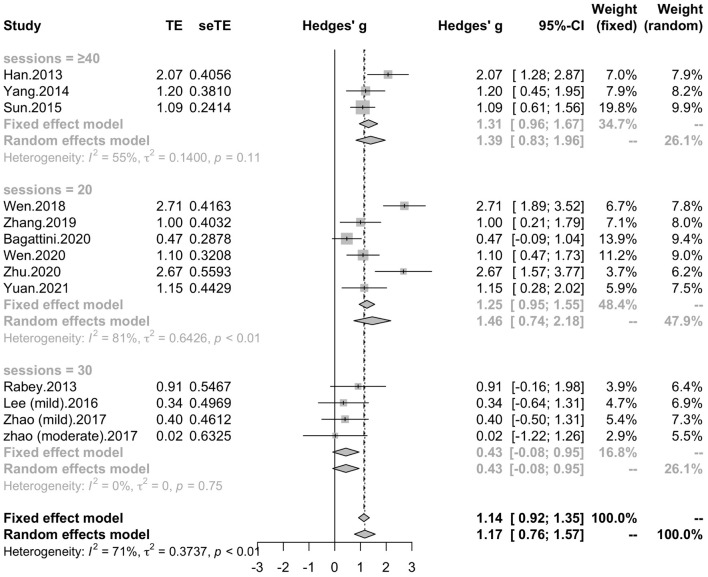
Forest plot: mean differences in session number subgroup with 95% CI.

**FIGURE 5:**
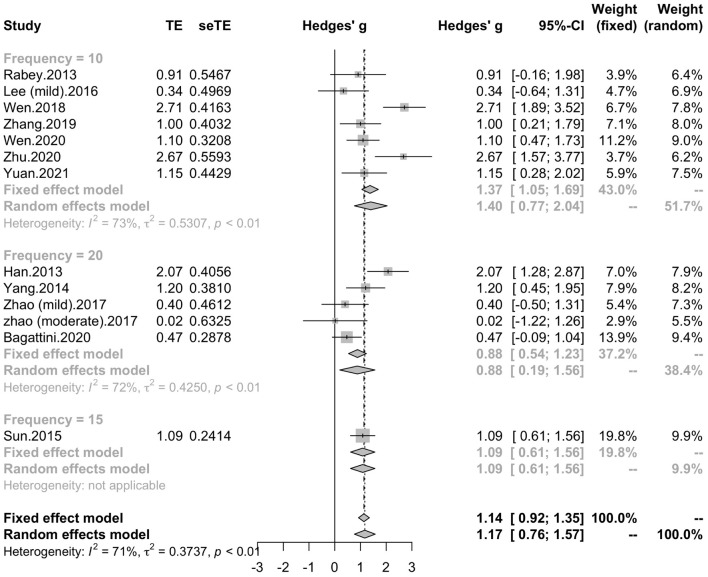
Forest plot: mean differences in frequency subgroup with 95% CI.

**FIGURE 6:**
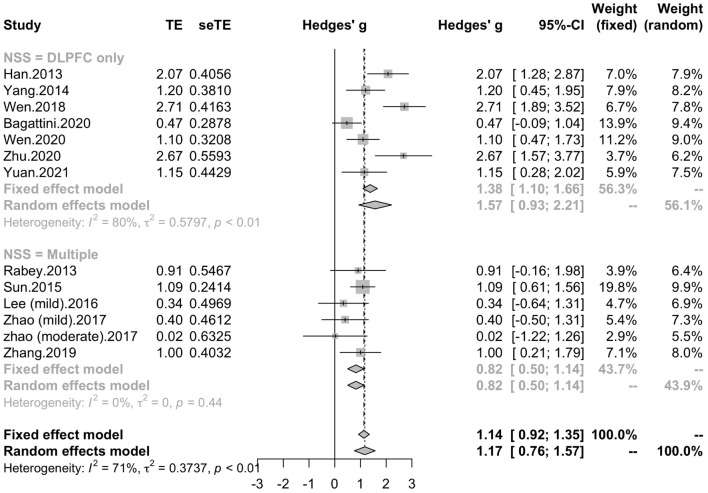
Forest plot: mean differences in stimulation site pattern subgroups with 95% CI. NSS, number of session site.

**FIGURE 7:**
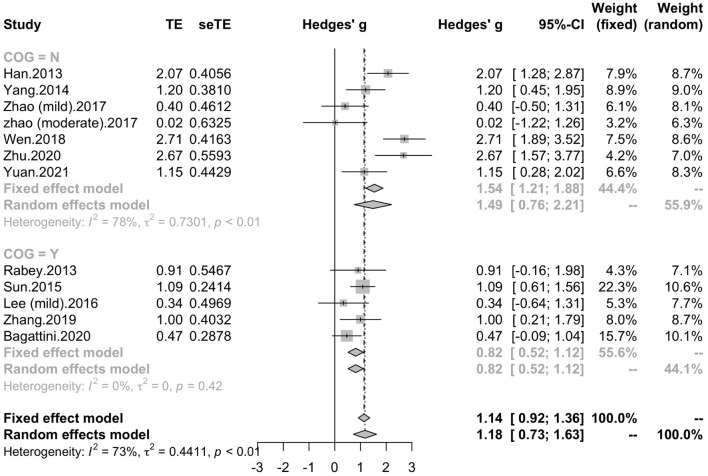
Forest plot: mean differences in cognitive training subgroups with 95% CI. COG, cognitive training.

To determine variables that may influence the cognitive outcomes, several subgroups were conducted. The subgroup analysis for session number of the stimulation (“20”, “30”, “≥ 40”) revealed a mean effect size of 1.46 (95% CI, 0.74 - 2.18) for trials with 20 sessions, a mean effect size of 0.43 (95% CI, -0.08 - 0.95) for trials with 30 sessions and a mean effect size of 1.39 (95% CI, 0.83 – 1.96) for the trials with ≥ 40 sessions (Figure 4). The effect size of subgroups of session number exhibited significant between-group difference (*p* = 0.0175). Analysis for frequency of the stimulation (“10 Hz”, “15 Hz”, “20 Hz”) revealed a mean effect size of 1.40 (95% CI, 0.77 – 2.04) for trials with frequency of 10 Hz, a mean effect size of 1.09 (95% CI, 0.61 – 1.56) for trials with frequency of 15 Hz and a mean effect size of 0.88 (95% CI, 0.19 - 1.56) for trials with frequency of 20 Hz (Figure 5). The subgroup analysis for the stimulation site pattern (“DLPFC only” vs “multiple site”) revealed a mean effect size of 1.57 (95% CI, 0.93 - 2.21) for trials with DLPFC as single site, and a mean effect size of 0.82 (95% CI, 0.50 - 1.14) for trials with multiple sites (Figure 6). The effect size of subgroups of stimulation site exhibited significant between-group difference (*p* = 0.0393). The subgroup analysis for combination with cognitive training (“Yes” vs “No”) revealed a mean effect size of 0.82 (95% CI, 0.52-1.12) for trials combining rTMS with cognitive training and a mean effect size of 1.49 (95% CI, 0.76-2.21) for trials not combining rTMS with cognitive training (Figure 7). The effect size of study *Zhao* et al. and study *Lee* et al. calculated with poststimulation evaluations didn’t change the results a lot. Only the mean effect size for trials with 30 sessions (0.61, 95% CI: 0.10 – 1.13) became significant and the difference between subgroups of session number became marginally significant (*p* = 0.069, Supplementary Table 1).

**FIGURE 8:**
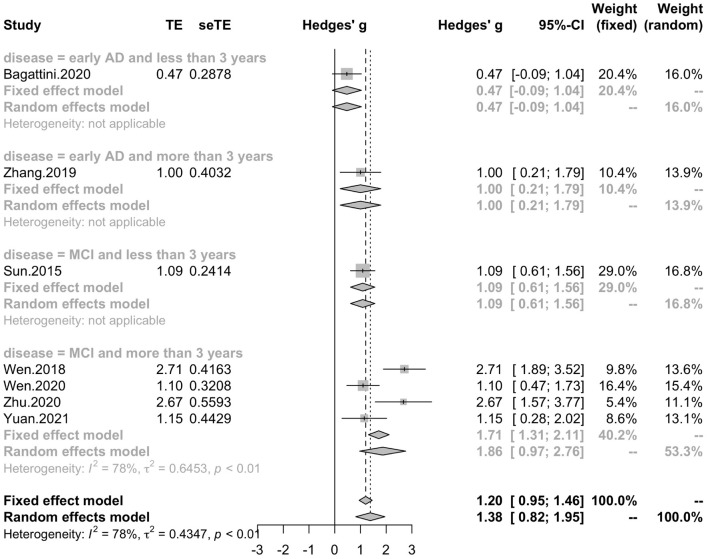
Forest plot: mean differences in disease characteristic subgroup with 95% CI.

**FIGURE 9:**
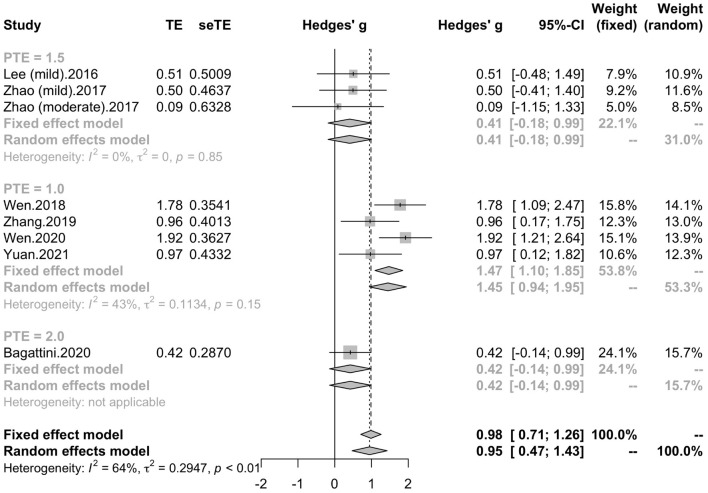
Forest plot: mean differences in post-treatment effect subgroup with 95% CI. PTE, post-treatment effect.

Seven studies reported the disease duration of participants. The subgroup analysis for the mean disease duration of the participants (“MCI and less than 3 years,” “MCI and more than 3 years,” “early AD and less than 3 years,” “early AD and more than 3 years) revealed a mean effect size of 1.86 (95% CI, 0.97 – 2.76) for trials with MCI patients whose disease durations were more than 3 years, a mean effect size of 1.09 (95% CI, 0.61 – 1.56) for trials with MCI patients whose disease durations were less than 3 years, a mean effect size of 1.00 (95% CI, 0.21 – 1.79) for trials with early AD patients whose disease duration were more than 3 years and a mean effect size of 0.47 (95% CI, -0.09 – 1.04) for trials with early AD patients whose disease duration were less than 3 years (Figure 8). Seven studies included eight trials reported post-treatment effect at different time points after the end of the treatment. The subgroup analysis for the post-treatment effect (“one month” vs “one and a half month” vs “two months”) revealed a mean effect size of 1.45 (95% CI, 0.94 – 1.95) for trials in which the post-treatment effect was assessed one month after the end of the treatment, a mean effect size of 0.41 (95% CI, -0.18 – 0.99) for trials in which the post-treatment effect was assessed one and a half months after the end of treatment and a mean effect size of 0.42 (95% CI, -0.14 – 0.99) for trials in which the post-treatment effect was assessed two months after the end of treatment (Figure 9). The effect size of subgroups of post-treatment effect exhibited significant between-group difference (*p* = 0.0075). With the effect size of study *Zhao* et al. and study *Lee* et al. calculated with poststimulation evaluations, the mean effect size for trials in which the post-treatment effect was assessed one and a half months after the treatment end (0.39, 95% CI: 0.09 – 0.70) became significant (Supplementary Table 1).

#### Memory, Executive Function and Attention, Language

**FIGURE 10:**
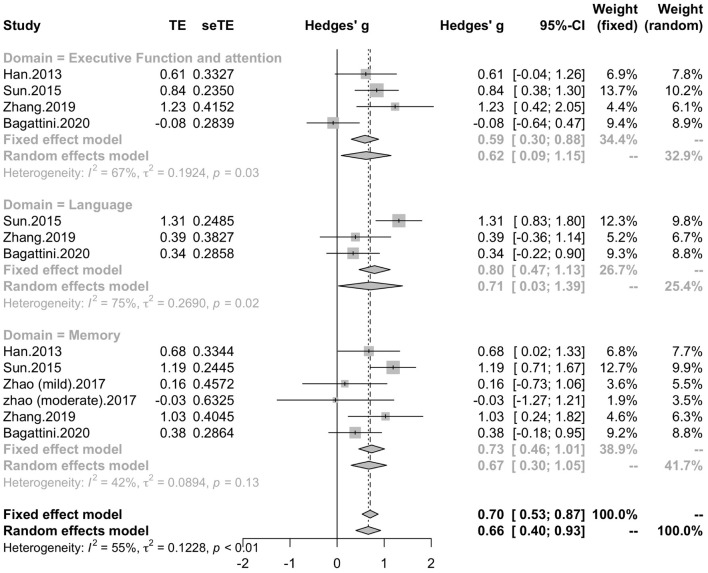
Forest plot: mean difference in effect of rTMS on different cognitive domain in patients with MCI or early AD with 95% CI.

Five studies included six trials reported the effect of rTMS on memory, while four studies reported the effect of rTMS on executive function and attention, and three studies reported the effect of rTMS on language. Subgroup analysis was conducted to test the effect of rTMS on these different domains, and a mean effect size of 0.67 (95% CI, 0.30 – 1.05) for trails of memory domains, a mean effect size of 0.62 (95% CI, 0.09 – 1.15) for trials of executive function and attention domains and a mean effect size of 0.71 (95% CI, 0.03 – 1.39) for trials of language (Figure 10). The effect size of study *Zhao* et al. and study *Lee* et al. calculated with poststimulation evaluations didn’t change the significance of effect size (Supplementary Table 1).

## Discussion

The current meta-analysis included thirteen studies with randomized, double-blind and controlled trials. These results added strong evidence for the efficacy of rTMS on cognitive improvement in patients with MCI and mild to moderate AD, not only in global cognitive function, but also in memory, language and executive function and attention. According to the subgroup analyses conducted to explore the proper stimulation patterns, most settings of rTMS parameters enhanced the global cognitive function, and the results revealed that rTMS protocols with stimulating frequency in 10 Hz and DLPFC as the stimulating site for 20 sessions would be able to produce benefit for the cognitive function. Besides, the post-treatment of rTMS has also be tested, and the result suggested a post-treatment effect on cognitive function for about one month and patients with MCI were likely to benefit more from the rTMS stimulation.

Consistent with the previous studies, the current results supported the benefit of rTMS on cognitive function in patients with MCI and AD, not only in the global cognitive outcomes, but also in the performance of memory, executive function and attention, and language. The impairments of memory, language and attention were cognitive manifestations of AD (Bracco et al., 2009; Jahn, 2013; Mueller et al., 2018; Malhotra, 2019). Recently, researchers have tested the effect of rTMS treatment on these cognitive domains and reported the cognitive benefit of rTMS on global cognitive function and sub-domains, like memory, language and executive function (Cotelli et al., 2011; Koch et al., 2018; Padala et al., 2018; Cui et al., 2020). It has been proposed that rTMS can modulate the cortical coactivation in the cognitive function-related brain areas (Cotelli et al., 2011). Previous studies demonstrated that rTMS of high frequency would facilitate cortical excitability and induce long-term potentiation (LTP) which has been implicated to be related to learning and memory (Motta et al., 2018; Di Lorenzo et al., 2019, 2020). The activation of the stimulation site would facilitate the connected larger-scale network and thus induce cognitive improvement in the multiple cognitive domains revealed by the current results.

Recent rTMS protocols have proposed the combination of rTMS and cognitive training, and our results also provide evidence to the improvement effect of the combination of rTMS and cognitive training on the cognitive performance (Bentwich et al., 2011; Das et al., 2019; Chu et al., 2020). It has been proposed that rTMS trials with cognitive training would combine the “exogenous” and “endogenous” stimulation to enhance neuroplasticity, in which the rTMS may be capable of pre-activating the initial state of neural system and the subsequent cognitive training would interact with the ongoing brain activation to potentiate or generalize the related neural impact (Miniussi and Rossini, 2011; Miniussi and Vallar, 2011; Bagattini et al., 2020). Thus, the cognitive training might be able to modulate the effect of rTMS, and might explain why the rTMS studies with cognitive training seemed to be more consistent than those studies without cognitive training. Besides, our current subgroup analysis of session number revealed that rTMS with more than 20 sessions might be able to produce cognitive enhancement in patients with MCI or early AD, which is consistent with previous studies (Lin et al., 2019; Wang et al., 2020; Jiang et al., 2021). However, the effect size of trials with 30 sessions seemed to be smaller than the effect size of trials with 20 session and with 40 sessions. The characteristics of trials have been checked and one alterative explanation was that the trials with 30 sessions had small sample sizes (most of them had less than 10 subjects for each group) and thus would limit the statistical power and resulted in lack of actual effects. Thus, trials with larger sample are in expectation to better test the effect of different rTMS parameters.

Different stimulation sites were used in the included studies of the current meta-analysis and subgroup analysis was conducted to test if rTMS treatment supported the cognitive improvement with the dorsolateral prefrontal cortex (DLPFC) used as the only stimulation brain area. Seven studies utilized left DLPFC or bilateral DLPFC as stimulation sites and other five studies utilized multiple brain regions. The results showed that those trials which stimulated DLPFC only (unilateral or bilateral) can improve the cognitive outcomes in patients with MCI and AD with an effect size significantly higher than those trials with multiple sites. DLPFC has been demonstrated as an important brain area subserving higher-level cognition and the its pathological change has been considered as a hallmark feature of AD from its early stage (Braak and Braak, 1991; Kumar et al., 2017). Besides, DLPFC has been considered as a key region playing important role in several large-scale brain networks, such as fronto-parietal network (FPN) and central executive network (CEN) (Agosta et al., 2012; Opitz et al., 2016). Considering its pivotal association with the cognitive impairment of AD, DLPFC has been used as stimulation site commonly in trials aiming to improving cognitive function in patients with AD. One study reported that rTMS at 5 Hz over left DLPFC would achieve similar cognitive improvement in AD patients compared to stimulation over multiple sites (Alcala-Lozano et al., 2018). The researchers proposed that left DLPFC connected with a variety of brain structures potentially involved in the pathophysiological progression in AD and thus the stimulation of DLPFC would also stimulate the areas engaged in the multi-site approach and produce equally positive outcomes (Alcala-Lozano and Garza-Villarreal, 2018). Although the results should be considered with caution due to its lack of a neuronavigator for the rTMS therapy, it still indicated the important role of DLPFC for rTMS stimulation. Besides, the efficacy of stimulation over unilateral or bilateral DLPFC is still under debate. A meta-analysis reported higher efficacy of right or bilateral DLPFC rTMS on cognitive outcomes over left DLPFC rTMS (Liao et al., 2015), and another study reported rTMS with a stimulation sequence of left DLPFC then right DLPFC was effective for cognitive outcomes (Ahmed et al., 2012). While the high-frequency rTMS over left/bilateral DLPFC has been reported to be effective on cognitive function, it has been reported that low-frequency rTMS of right DLPFC enhanced recognition memory in eight subjects with MCI (Turriziani et al., 2012). It has been proposed that the inhibition of the right DLPFC might modulated the activity of the dysfunctional network and thus restoring an adaptive equilibrium in MCI. Thus, further studies with DLPFC as stimulation site combining with multiple-modality data are needed to explore the efficacy of rTMS stimulation over DLPFC and its underlying mechanism.

How long would the cognitive benefit of rTMS in patients of MCI and AD prolong is another important issue that people care about. The current meta-analysis showed a significant effect size for the trials which tested the lasting effect one month after the treatment end. It has been reported that rTMS is able to induce long-lasting changes of cortical excitability (Nardone et al., 2014), and whether these changes would last after stopping the rTMS is still under exploration. Several studies utilized multi-timepoint assessments to test the lasting effect of rTMS (Lee et al., 2016; Nguyen et al., 2017; Bagattini et al., 2020). *Cotelli et al.* has reported that with a 2-week rTMS stimulation over left parietal cortex, aMCI patients improved their accuracy in an association memory task and such improvement remained significant 24 weeks after stimulation began (Cotelli et al., 2012). However, there are limitations that some studies lacked of data in the control group and cannot provide a better evaluation for the effect of rTMS. The current results suggested that the alteration of brain mechanism induced by rTMS might still support the improvement of global cognitive function for about one month even without the rTMS treatment when compared to the control group. Considering the small number of trials included in each subgroup, particularly for post-treatment effect assessed two months after the treatment end, the current results should be interpreted with caution and more studies still in need to provide evidence for the long-lasting effect of rTMS.

Except the post-treatment effect of rTMS, we also tested whether pre-treatment condition of patients would influence the effect of rTMS on the global cognitive function. Our results revealed that patients with lighter clinical manifestation and longer disease duration seemed to benefit more from rTMS treatment. It might suggest that patients with less cognitive impairment, or degenerating slower (have maintained in such stage for a longer time), might reflect a better pre-treatment brain mechanism compared to the patients whose symptoms worsened to a similar level in shorter duration, and thus would enable rTMS to be more efficient. Previous studies have reported the difference of LTP introduced by rTMS among different stage of AD, while the induced LTP has been regarded as the pivotal mechanism in which the rTMS treatment can support the cognitive benefit in AD patients (Maeda et al., 2000; Tegenthoff et al., 2005; Di Lorenzo et al., 2019, 2020). rTMS intervention has also be reported to be more marked cognitive benefit in patients at an early state of AD (Rutherford et al., 2015). It has also been reported that the variability of rTMS induced cognitive after-effects would be influenced by gray mater atrophy of AD-related brain regions (Anderkova et al., 2015). Current results should be interpreted with caution due to the small number of trials, however, it still called attention to applying intervention earlier and emphasized the importance to explore the biomarkers of pre-treatment brain mechanism for rTMS in developing better and individual-specific intervention protocol.

## Limitation

A few limitations should be considered when interpreting the findings of the current study. First, with constrained inclusion criteria, the number of trials included in the meta-analysis is limited, and although Hedges’ g was used as SMD, the sample sizes of the included studies were small, which might limit the statistical power to detect the effects of rTMS on cognitive function in patients with MCI or early AD. Second, we can’t assess the change of treatment relative to the baseline for all the studies, and according to the effect sizes of the two studies (Lee et al., 2016; Zhao et al., 2017), we found that there is a little difference between effect sizes calculated with change relative to the baseline and those calculated with poststimulation outcomes, and thus induced a small shift of the effect size in some subgroup analyses. The evaluation of the rTMS cognitive effect would be more reliable with the effect size calculated by the change relative to the baseline, but the practical analysis was limited by the difficulty to assess the data. Third, optimal rTMS parameters remained unclear because of the relatively high heterogeneity of the included studies in the subgroup analyses of stimulation parameters (frequency, session number, stimulation site). Further randomized, double-blinded and controlled rTMS studies focusing on MCI or early AD are in expectation to be more sophisticated designed and better result-reporting.

## Conclusion

In conclusion, our meta-analysis provided evidence that rTMS therapy in patients with MCI or early AD can significantly improve not only global cognitive ability, but also memory, executive function and language when compared to the control group. Most settings of rTMS parameter can significantly improve the global cognitive function and the results showed that rTMS protocol with frequency of 10 Hz and DLPFC as stimulation site for continuous 20 sessions would be capable to produce cognitive benefit. The cognitive benefit of rTMS treatment can last for about one month after the end of treatment. Patients with earlier course of AD would be more likely to benefit more from rTMS treatment. Current study provided critical information for optimal parameters of rTMS therapy and indicate the importance to consider the pre-treatment physiological condition of patients when evaluated the effect of rTMS therapy in patients with MCI and early AD. Further researches with larger sample sizes and better experiment design were crucially needed to identify the optimal parameters of rTMS intervention on cognition of AD patients.

## Author Contributions

YK conceived and supervised the study. YX, LN, and WZ screened literature and data extraction. YX and ZK performed statistical analyses. YX, ZK, and YK wrote the manuscript. YL edited the manuscript. All authors contributed to the article and approved the submitted version.

## Conflict of Interest

The authors declare that the research was conducted in the absence of any commercial or financial relationships that could be construed as a potential conflict of interest.

## Publisher’s Note

All claims expressed in this article are solely those of the authors and do not necessarily represent those of their affiliated organizations, or those of the publisher, the editors and the reviewers. Any product that may be evaluated in this article, or claim that may be made by its manufacturer, is not guaranteed or endorsed by the publisher.
