# Supplementary material for: Cognitive Enhancement of Repetitive Transcranial Magnetic Stimulation in Patients With Mild Cognitive Impairment and Early Alzheimer’s Disease: A Systematic Review and Meta-Analysis
Source: Front Cell Dev Biol. 2021 Sep 10;9:734046. doi: 10.3389/fcell.2021.734046 (PMC8461243; doi:10.3389/fcell.2021.734046)
Supplement: Supplementary file 1 [file Data_Sheet_1.docx]

# Table

## Supplementary Table 1

| Subgroups | Number of Trials | SMD (95% CI) | tau |
| --- | --- | --- | --- |
| *Frequency* |  |  |  |
| 10 Hz | 7 | 1.40 (0.76, 2.04) | 0.74 |
| 15 Hz | 1 | 1.09 (0.62, 1.56) | \ |
| 20 Hz | 5 | 1.02 (0.40, 1.64) | 0.56 |
| *Session Number* |  |  |  |
| 20 | 6 | 1.46 (0.74, 2.18) | 0.80 |
| 30 | 4 | 0.61 (0.10, 1.13) | 0 |
| ≥ 40 | 2 | 1.39 (0.83, 1.96) | 0.37 |
| *Stimulation Pattern* |  |  |  |
| DLPFC only | 4 | 1.55 (0.59, 2.52) | 87 |
| Multiple | 6 | 0.89 (0,57, 1.21) | 0 |
| *Cognitive Training** |  |  |  |
| Yes | 5 | 0.81 (0.51, 1.11) | 0.028 |
| No | 7 | 1.60 (0.97, 2.23） | 0.71 |
| *Disease Characteristic* |  |  |  |
| MCI and more than 3 years | 4 | 1.86 (0.97, 2.76) | 0.65 |
| MCI and less than 3 years | 1 | 1.09 (0.61, 1.56) | \ |
| Early AD and more than 3 years | 1 | 1.00 (0.21, 1.79) | \ |
| Early AD and less than 3 years | 1 | 0.47 (-0.09, 1.04) | \ |
| *Post-treatment Effect** |  |  |  |
| One month | 4 | 1.45 (0.94, 1.95) | 0.34 |
| One and a half month | 3 | 0.39 (0.09, 0.70) | 0 |
| Two months | 1 | 0.42 (-0.14, 0.99) | \ |
| *Cognitive Domains* |  |  |  |
| Executive function and attention | 4 | 0.62 (0.09, 1.15) | 0.44 |
| Language | 3 | 0.71 (0.03, 1.39) | 0.52 |
| Memory | 6 | 0.67 (0.29, 1.05) | 0.30 |

*, *p < 0.05 for subgroup difference.*

# Figure

## Supplementary Figure 1

Forest plot: mean differences in effect of rTMS on different cognitive domain in patients with MCI or early AD with 95% CI.
